# Supplementary material for: Dynamics of Cytokine, SARS-CoV-2-Specific IgG, and Neutralizing Antibody Levels in COVID-19 Patients Treated with Convalescent Plasma
Source: Diseases. 2023 Aug 30;11(3):112. doi: 10.3390/diseases11030112 (PMC10527804; doi:10.3390/diseases11030112)
Supplement: Supplementary file 1 [file diseases-11-00112-s001.zip › diseases-2554299-supplementary.pdf]

**Supplementary data**

We provide inter- and intra-assay CoV, specificity, and sensibility of the kits used for antibody and cytokine evaluations.

The Abbott SARS-CoV-2 IgG assay showcases an exceptional diagnostic specificity of 100.00% (95% CI: 95.89-100.00) in both inter- and intra-assay assessments. Furthermore, it displays a noteworthy sensitivity, with a diagnostic sensitivity of 99.60% (95% CI: 98.98-99.98). In the case of the Elecsys anti-SARS-CoV-2 assay, it demonstrates consistent inter-assay and intra-assay coefficients of variation (CoV), underscoring its robust performance. Significantly, the assay achieves a diagnostic specificity of 99.95% (99.87–99.99), emphasizing its precision in identifying true negative cases. Concurrently, the assay exhibits a substantial diagnostic sensitivity of 97.92% (95.21–99.32), highlighting its efficacy in detecting true positive cases.

Table S1. For intra- and inter-assay CV%, sensitivity, and assay working ranges for each platform

| | Assay Working Ranges, pg/ml | | Assay Sensitivity, pg/ml | Intra-Assay %CV | Inter-Assay %CV |
|---|---|---|---|---|---|
| Analyte | LLOQ | ULOQ | LOD | | |
| IFN-α2 | 0.95 | 15,569 | 0.46 | 3.3 | 4.4 |
| IL-1β | 0.29 | 4,672 | 0.24 | 3.6 | 3.2 |
| IL-6 | 0.38 | 6,244 | 0.34 | 2.2 | 3.0 |
| IL-10 | 1.06 | 17,427 | 0.69 | 2.3 | 3.4 |
| IFN-β | 1 | 3,069 | 0.42 | 2.95 | 20.78 |
| sIL-6Rα | 17 | 36,429 | 1.8 | 2.02 | 10.51 |

CV, coefficient of variation; LLOQ, lower limit of quantitation; LOD, limit of detection; ULOQ, upper limit of quantitation.
